# Risk of Psychiatric Disorders following Polycystic Ovary Syndrome: A Nationwide Population-Based Cohort Study

**DOI:** 10.1371/journal.pone.0097041

**Published:** 2014-05-09

**Authors:** Jeng-Hsiu Hung, Li-Yu Hu, Shih-Jen Tsai, Albert C. Yang, Min-Wei Huang, Pan-Ming Chen, Shu-Li Wang, Ti Lu, Cheng-Che Shen

**Affiliations:** 1 Department of Obstetrics and Gynecology, Taipei Tzu Chi Hospital, Buddhist Tzu Chi Medical Foundation, Taipei, Taiwan; 2 School of Medicine, Tzu Chi University, Hualien, Taiwan; 3 Department of Psychiatry, Kaohsiung Veterans General Hospital, Kaohsiung, Taiwan; 4 Department of Psychiatry, Taipei Veterans General Hospital, Taipei, Taiwan; 5 Center for Dynamical Biomarkers and Translational Medicine, National Central University, Chungli, Taiwan; 6 Department of Psychiatry, Chiayi Branch, Taichung Veterans General Hospital, Chiayi, Taiwan; 7 Department of Psychiatry, Yuanshan Branch, Taipei Veterans General Hospital, Yilan, Taiwan; 8 Department of Dental Laboratory Technology, entral Taiwan University of Science and Technology, Taichung, Taiwan; 9 Department of information magagement, National Chung-Cheng University, Chiayi, Taiwan; 10 Institute of Public Health, National Yang-Ming University, Taipei, Taiwan; 11 School of Medicine, National Yang-Ming University, Taipei, Taiwan; Imperial College London, United Kingdom

## Abstract

**Background:**

Polycystic ovary syndrome (PCOS) is one of the most common endocrine disorders among women of reproductive age. A higher prevalence of psychiatric comorbidities, including depressive disorder, anxiety disorder, and bipolar disorder has been proved in patients with PCOS. However, a clear temporal causal relationship between PCOS and psychiatric disorders has not been well established.

**Objective:**

We explored the relationship between PCOS and the subsequent development of psychiatric disorders including schizophrenia, bipolar disorder, depressive disorder, anxiety disorder, and sleep disorder.

**Methods:**

We identified patients who were diagnosed with PCOS by an obstetrician-gynecologist in the Taiwan National Health Insurance Research Database. A comparison cohort was constructed of patients without PCOS who were matched according to age and sex. The occurrence of subsequent new-onset psychiatric disorders was evaluated in both cohorts based on diagnoses made by psychiatrists.

**Results:**

The PCOS cohort consisted of 5431 patients, and the comparison cohort consisted of 21,724 matched control patients without PCOS. The incidence of depressive disorder (hazard ratio [HR] 1.296, 95% confidence interval [CI] 1.084–.550), anxiety disorder (HR 1.392, 95% CI 1.121–1.729), and sleep disorder (HR 1.495, 95% CI 1.176–1.899) were higher among the PCOS patients than among the patients in the comparison cohort. In addition, a higher incidence of newly diagnosed depressive disorder, anxiety disorder, and sleep disorder remained significantly increased in all of the stratified follow-up durations (0–1, 1–5, ≥5 y).

**Conclusions:**

PCOS might increase the risk of subsequent newly diagnosed depressive disorder, anxiety disorder, and sleep disorder. The risk of newly diagnosed bipolar disorder, which has often been reported in the literature to be comorbid with PCOS, was not significantly elevated.

## Introduction

Polycystic ovary syndrome (PCOS) is one of the most common endocrine disorders among women and affects 5% to 10% of women of reproductive age [1]. PCOS is a complex illness that is characterized by irregular menses, excessive amounts of androgenic hormones, and polycystic ovaries [2]. Recently, it has been postulated that PCOS is linked to an increased risk for cardiovascular diseases [3] and type 2 diabetes [4] in affected women. Therefore, PCOS must be accepted not only as a reproductive problem but also as a severe metabolic disease that carries crucial health risks as age increases.

In addition, interest in the psychiatric aspects of PCOS has grown. According to previous studies, 56.9% of women with PCOS have at least one psychiatric disorder [5]–[7], and higher incidences of psychiatric disorders have been observed in women with PCOS, particularly depressive disorder, bipolar disorder, and anxiety disorder [8]–[10]. Furthermore, a study reported that these comorbid psychiatric problems affect the quality of life of PCOS patients [11].

Although the above mentioned research has provided insight to the association between PCOS and comorbid psychiatric disorders, most of these study results are based on cross-sectional study design and lack a longitudinal perspective. In addition, in these studies, psychiatric disorders were often evaluated using rating scales, such as Mini International Neuropsychiatric Interview, the Beck depression inventory or the Hamilton depression rating scale, rather than diagnosis by a psychiatrist. Furthermore, the small sample size of most of these studies prevents generalization. Evidence has shown that hormonal change, which is a typical clinical presentation of PCOS, can increase the susceptibility of women to psychiatric conditions [12]–[14]. While considerable attention has been paid in the past to research issues related to the psychiatric comorbidities among PCOS patients, little empirical evidence has been found to support a clearer association between the PCOS and subsequent psychiatric disorders.

In response to the lack of national data and few longitudinal studies concerning the association between PCOS and the subsequent risk of psychiatric disorders, and based on the hypothesis that PCOS might have a higher risk for developing subsequent psychiatric disorders, we designed a nationwide population-based retrospective cohort study to investigate the possible link between these 2 illnesses.

## Patients and Methods

### Data Sources

Instituted in 1995, the National Health Insurance (NHI) program is a mandatory health insurance program that offers comprehensive medical care coverage, including outpatient, inpatient, emergency, and traditional Chinese medicine, to all residents of Taiwan, with a coverage rate of up to 98% [15]. The NHI Research Database (NHIRD) contains comprehensive information regarding clinical visits, including prescription details and diagnostic codes based on the A code and International Classification of Diseases, Ninth Revision, Clinical Modification (ICD-9-CM). The NHIRD is managed and publicly released by the National Health Research Institutes (NHRI) for research purposes, and confidentiality is maintained according to the directives of the Bureau of NHI. The data source for our study was the Longitudinal Health Insurance Database 2005 (LHID 2005), which is a dataset of NHIRD. Data for the LHID was collected by systematically and randomly sampling from the NHIRD; the database included the data of one million individuals. The NHRI of Taiwan reports that there were no significant differences in gender distribution, age distribution, or average insured payroll-related amount between the patients in the LHID and those in the original NHIRD [16].

### Ethics Statement

The Institutional Review Board of Taipei Veterans General Hospital approved this study (2013-03-035AC). Written consent from the study patients was not obtained, because the NHI dataset consists of deidentified secondary data for research purposes and the Institutional Review Board of Taipei Veterans General Hospital issued a formal written waiver for the need for consent.

### Study Population

Using data extracted from the LHID 2005, we conducted a retrospective cohort study of patients who were newly diagnosed with PCOS by an obstetrician-gynecologist between January 1, 2000 and December 31, 2008. The patients with PCOS were defined as ICD-9-CM code: 256.4. We excluded patients who were diagnosed with PCOS (A code: A189; ICD-9-CM code: 256.4) between January 1, 1996, and December 31, 1999. We also excluded patients who were diagnosed with psychiatric disorders (A codes: A210-A219; ICD-9-CM codes: 290-319) before they were diagnosed with PCOS. For each PCOS patient included in the final cohort, 4 age- and sex-matched control patients without psychiatric disorder were randomly selected from the LHID 2005. All PCOS and control patients were observed until diagnosed with schizophrenia (ICD-9-CM code: 295), depressive disorder (ICD-9-CM codes: 296.2, 296.3, 300.4, and 311), bipolar disorder (ICD-9-CM codes: 296.0, 296.1, 296.4, 296.5, 296.6, 296.7, 296.8, 296.80, and 296.89), anxiety disorder (ICD-9-CM codes: 300.0, 300.2, 300.3, 308.3, and 309.81), or sleep disorder (ICD-9-CM codes: 780.5, 307.4 [excluding 780.51, 780.53, 780.57]), or until death, withdrawal from the NHI system, or December 31, 2009. The primary clinical outcomes assessed were psychiatrist-diagnosed schizophrenia, depressive disorder, bipolar disorder, anxiety disorder, and sleep disorder. Common comorbidities including hypertension, diabetes mellitus, dyslipidemia, congestive heart failure, chronic pulmonary diseases, coronary artery diseases, cerebrovascular diseases, and malignancies were also compared between PCOS and control patients.

### Statistical Analysis

The occurrence of newly diagnosed schizophrenia, depressive disorder, bipolar disorder, anxiety disorder, or sleep disorder in the PCOS and control patients were considered as the primary outcome in this study. We first compared the distribution of demographic characteristics between the PCOS and control patients by using the independent *t*-tests and chi-squared test. To investigate potential surveillance bias, subgroups were stratified according to the duration since PCOS diagnosis. In addition, a Cox proportional-hazards regression model was constructed to calculate the hazard ratio (HR) of schizophrenia, depressive disorder, bipolar disorder, anxiety disorder, and sleep disorder of the PCOS cohort and control cohort.

The SAS statistical software for Windows, Version 9.3 (SAS Institute, Cary, NC, USA), was used for data extraction, computation, linkage, processing, and sampling. All other statistical analyses were performed using the SPSS statistical software for Windows, Version 20 (IBM, Armonk, NY, USA). The results of comparisons with a *P* value less than .05 were considered to indicate a statistically significant relationship.

## Results

Our study sample comprised 5431 PCOS patients and 21,724 control patients without PCOS. The comparisons of the demographic and clinical variables between the PCOS and control patients are presented in Table 1. The median age of the patients was 26.66 years (interquartile range, 22.27 to 31.36 y) and the median follow-up duration was 5.07 years (interquartile range, 3.02 to 7.21 y). A higher percentage of patients with PCOS were observed in the group of people aged 20–39 years. Chronic pulmonary diseases and dyslipidemia were the 2 most common observed comorbidities in PCOS and control patients. Baseline difference in comorbidities demonstrated higher prevalence of chronic pulmonary diseases, dyslipidemia, diabetes mellitus, hypertension, and congestive heart failure among the PCOS patients.

**Table 1:** Characteristics of patients with polycystic ovarian syndrome (PCOS) and control subjects.

	PCOS	Control	*P* values
No.	5,431	21,724	>0.999
Age (years)^a^	26.66 (22.27–31.36)	26.66 (22.27–31.36)	0.940
Distribution of age			
0–19	782	3,128	0.996
20–39	4,400	17,600	
40–59	249	996	
≥60	0	0	
Comorbidities			
Hypertension	109 (2.00)	239 (1.10)	<0.001*
Diabetes mellitus	180 (3.31)	390 (1.80)	<0.001*
Dyslipidemia	241 (4.44)	467 (2.15)	<0.001*
Coronary artery disease	3 (0.06)	6 (0.03)	0.317
Congestive heart failure	12 (0.21)	22 (0.10)	0.026*
Cerebrovascular disease	35 (0.64)	107 (0.49)	0.165
Chronic pulmonary disease	778 (14.33)	2509 (11.55)	<0.001*
Malignancy	9 (0.17)	57 (0.26)	0.196
Follow-up, years^a^	5.07 (3.02–7.21)	5.06 (3.02–7.21)	0.940
Newly diagnosed psychiatric disorders, N (%)			
Schizophrenia	11 (0.20)	53 (0.24)	0.573
Bipolar disorder	12 (0.22)	49 (0.23)	0.949
Depressive disorder	159 (2.93)	492 (2.26	0.004*
Anxiety disorder	110 (2.03)	317 (1.46)	0.003*
Sleep disorder	95 (1.75)	251 (1.16)	<0.001*

a Median (interquartile range);

*Statistical significance.

During the follow-up period, 387 (7.13%) PCOS patients and 159 (2.93%) control patients were diagnosed with psychiatric disorders (*P*<.001). The most common subsequent psychiatric disorders in the patients with PCOS were depressive disorder in 159 patients (2.93%), anxiety disorder in 110 patients (2.03%), and sleep disorder in 95 patients (1.75%). Overall, significantly higher incidences of depressive disorder (*P* = .004), anxiety disorder (*P* = .003), and sleep disorder (*P*<.001) were observed in the PCOS patients than in the control patients.

In addition, a Cox proportional-hazards regression analysis was conducted to calculate the HR of the newly diagnosed psychiatric disorders for the PCOS patients compared with the matched controls (Table 2). The results indicated that the patients with PCOS exhibited a markedly higher risk for subsequent depressive disorder (HR 1.296, 95% confidence interval [CI] 1.084–1.550), anxiety disorder (HR 1.392, 95% CI 1.121–1.729), and sleep disorder (HR 1.495, 95% CI 1.176–1.899).

**Table 2:** Hazard ratios of time until psychiatric disorders between patients with polycystic ovarian syndrome (PCOS) and control subjects during a ten-year follow-up period.

	HR	95% CI	*P* value
Schizophrenia	0.83	0.433–1.588	0.573
Bipolar disorder	0.979	0.520–1.840	0.946
Depressive disorder	1.296	1.084–1.550	0.004*
Anxiety disorder	1.392	1.121–1.729	0.003*
Sleep disorder	1.495	1.176–1.899	0.001*

HR hazard ratio; CI confidence interval;

*Statistical significance.

Furthermore, a subanalysis based on the duration of follow up revealed that most of the depressive disorder, anxiety disorder, and sleep disorder developed beyond the first year following a PCOS diagnosis and that the risk of these newly diagnosed psychiatric disorders remained significantly elevated when the patients were stratified according to follow-up duration (0–1, 1–5, ≥5 y). The results of the subanalysis are summarized in Table 3.

**Table 3:** Number of newly diagnosed psychiatric disorders between patients with polycystic ovarian syndrome (PCOS) and control subjects which was stratified by follow-up duration.

Follow-up duration (years)	Depressive disorder (N)			Anxiety disorder (N)			Sleep disorder (N)		
	PCOS	Control	*P* values	PCOS	Control	*P* values	PCOS	Control	*P* values
0–1	36	79	<0.001*	12	35	<0.001*	14	26	<0.001*
1–5	90	300	<0.001*	62	195	<0.001*	59	154	<0.001*
≥5	33	113	<0.001*	36	87	<0.001*	22	71	<0.001*

*Statistical significance.

## Discussion

This is the first population-based retrospective study to examine PCOS and the risk of subsequent newly onset psychiatric disorders. The major finding of our study is the significantly higher incidence of subsequent depressive disorder, anxiety disorder, and sleep disorder, but not schizophrenia and bipolar disorder, among patients with PCOS.

Several studies have revealed a higher prevalence of psychiatric disorders, particularly depressive disorder, anxiety disorder, and bipolar disorder, in patients with PCOS [8]–[10]. However, caution in interpreting these results is warranted due to several methodological constraints such as relatively small case numbers, lack of longitudinal observation, absence of demographically matched control group with PCOS, and use of some rating scales or self-report screens which are not corroborated by a diagnostic interview. In addition, these studies focused on the association between PCOS and psychiatric comorbidities; therefore, unanswered questions remain regarding the association between PCOS and new-onset psychiatric disorders. In our study design, we excluded previous psychiatric disorders among both study groups and investigated the incidence of newly diagnosed psychiatric disorders based on the hypothesis that PCOS might be a risk factor for developing subsequent psychiatric disorders. In addition, to ensure that the PCOS diagnoses were valid, only patients who were diagnosed with PCOS by an obstetrician-gynecologist were included in the PCOS cohort. Similarly, we included only psychiatrist-diagnosed psychiatric disorders in our analysis.

Several studies have confirmed that depression and anxiety are the most common comorbidities in patients with PCOS [6], [17], [18]. Our study indicated that PCOS might be a risk factor for subsequent depressive and anxiety disorders. The factors that play a role in the development of anxiety in women with PCOS remain undetermined. Certain studies have reported that hirsutism [19] and acne [20] are associated with anxiety, whereas another study did not observe this association [21]. Numerous possible explanations can be provided for the result regarding the increased risk of depressive disorder in PCOS patients. First, higher serum levels of androgens have been implicated in the incidence of psychological disturbances among PCOS patients [8]. Hirsutism and acne resulting from hyperandrogenism in patients with PCOS are strongly associated with a person' dissatisfaction with their body and depression [22], indicating a link between hyperandrogenism and depression among women with PCOS. However, no consistent evidence has been provided supporting the finding that hyperandrogenism directly increases the risk of developing depressive disorder [23]–[25]. Second, insulin resistance [26] and obesity [27]–[29], which are common symptoms in patients with PCOS, have been noted to be related to depressive symptoms. Insulin resistance affects 50%–70% [30] of women with PCOS and the effect of obesity on insulin resistance is additive to that of PCOS [3]. Studies have demonstrated insulin resistance in women with PCOS might lead to a number of comorbidities including hypertension [4], type 2 diabetes [31] and dyslipidemia [32], [33]. Evidence for higher prevalence of physical comorbidities among PCOS patients is consistent with our study result. Therefore, another possible explanation of the higher risk of depressive disorder is PCOS-related comorbidities. However, the underlying mechanisms of the relationship are currently poorly understood. Lastly, studies have indicated that depressive disorders among PCOS patients might be caused by psychological stresses associated with reduced fertility or assisted reproductive treatment [34].

Evidence has shown that schizophrenia is not more highly prevalent among the PCOS patients than patients without PCOS [5], [35]. In our study, no significantly increased risk of subsequent schizophrenia was observed after a diagnosis of PCOS. Regarding the relationship between PCOS and bipolar disorder, previous studies have reported that PCOS is associated with bipolar disorder. However, our results indicated that PCOS did not increase the risk of subsequent newly diagnosed bipolar disorder. These 2 discrete findings might be explained by the prescription of valproic acid, which is a commonly used medication in patients with bipolar disorder or epilepsy. The increased prevalence of PCOS associated with valproic acid prescription was observed in both women with epilepsy and women with bipolar disorders [36]. Although Klipstein et al. have investigated the hypothesis that an intrinsic association between PCOS and bipolar disorder exists independent of valproic acid use [9], it is crucial to emphasize that the interpretations of their study results are limited by the possible selection bias, lack of a demographically matched control group without PCOS, and use of a self-report screen alone to screen for, rather than diagnose, bipolar disorder. Therefore, additional studies with large sample size and suitable study design should be conducted to investigate the association between PCOS and the risk of bipolar disorder.

Moreover, our study indicated that patients with PCOS exhibit an increased risk for developing new-onset sleep disorders. We hypothesized that this risk is linked to 2 possible reasons. First, the prevalence of obstructive sleep apnea (OSA) was higher in PCOS patients [37], and the mechanisms might be associated with the obesity caused by PCOS-induced metabolic abnormalities [38]. Second, regardless of OSA, studies have shown that the sleep disorders in patients with PCOS might be related to raised nighttime urinary melatonin levels, which are associated with lower sleep quality [39]. In addition, other preliminary hypotheses for sleep disorder among PCOS patients, including the impact of insulin resistance and hyperandrogenemia, have been postulated in the literature [40].

In the present study, we conducted a subgroup analysis stratified according to the duration between the diagnosis of PCOS and new-onset psychiatric disorders. The results indicated that incident depressive disorder, anxiety disorder, and sleep disorder were increased in the first year after a diagnosis of PCOS. One possible explanation for this result is surveillance bias [41]. Patients with PCOS are likely to exhibit a higher frequency of outpatient visits than the general population, leading to an earlier diagnosis of psychiatric disorders. When excluding data from patients for whom PCOS and psychiatric disorders were diagnosed within 1 year, a higher incidence of newly diagnosed depressive disorder, anxiety disorder, and sleep disorder remained significantly increased. Hence, concluding that the increased risk of depressive disorder, anxiety disorder, and sleep disorder in PCOS patients in the current study was not due only to surveillance bias is reasonable.

Our study is one of the few nationwide retrospective studies that have examined PCOS as a risk factor for the development of subsequent psychiatric disorders. However, it has several limitations inherent to the use of claims databases that should be considered. First, the causes of psychiatric disorders are generally complex and vary depending on the individual. It has been found that many psychological and environmental factors can all contribute to the development of psychiatric disorders. We acknowledged that several important demographic variables were unavailable in the medical care database, such as family history of psychiatric disorders, stressful life situations, interpersonal relationships and lifestyle. Moreover, psychiatric disorders are often a result of a combination of several different factors rather than just a single factor. Second, the consensus regarding to the PCOS diagnosis criteria is still lacking. In 2004, the National Institute of Health recognized PCOS as a syndrome and no single criterion sufficient for diagnosis. In our study, patients who were coded with 256.4 not only have polycystic ovaries but also have other symptoms including menstrual disorders, infertility, and signs of androgen excess such as acne, hirsutism or androgenic alopecia. Moreover, we included obstetrician-gynecologist diagnosed PCOS only to increase the accuracy of diagnosis, because almost all PCOS patients included in Taiwan received gynecologic ultrasound and hormone tests to confirm the diagnosis. However, data on the severity of PCOS were limited. The effects of the severity of PCOS on the development of subsequent psychiatric disorders require further investigation. Third, the duration of the follow-up period in our study might have been insufficient for detecting late-onset psychiatric disorders. Thus, future studies with longer follow-up periods are required to more clearly elucidate the long-term risk of psychiatric disorders among patients with PCOS.

In conclusion, this nationwide cohort study indicated an increased risk of depressive disorder, anxiety disorder, and sleep disorder among PCOS patients. The risk of newly diagnosed bipolar disorders, which has often been reported in the literature to be comorbid with PCOS, was not significantly elevated. Prospective studies, particularly those with additional patient-level data, are warranted to confirm our findings.
